# Risk of Depression and Anxiety in Adults With Cerebral Palsy

**DOI:** 10.1001/jamaneurol.2018.4147

**Published:** 2018-12-28

**Authors:** Kimberley J. Smith, Mark D. Peterson, Neil E. O’Connell, Christina Victor, Silvia Liverani, Nana Anokye, Jennifer M. Ryan

**Affiliations:** 1 Department of Psychological Sciences, Faculty of Health and Medical Sciences, University of Surrey, Guildford, United Kingdom; 2 Ageing Studies Research Group, Institute for Environment and Societies, Brunel University London, Uxbridge, Middlesex, United Kingdom; 3 Department of Physical Medicine and Rehabilitation, University of Michigan, Ann Arbor; 4 Department of Clinical Sciences, Brunel University London, Uxbridge, United Kingdom; 5 School of Mathematical Sciences, Queen Mary’s University London, London, United Kingdom; 6 Department of Epidemiology and Public Health Medicine, Royal College of Surgeons in Ireland, Dublin, Ireland

## Abstract

**Importance:**

Cerebral palsy (CP) is considered a pediatric condition despite most individuals with CP living into adulthood. Thus, there is a lack of evidence in adults with CP, which includes a paucity of research examining mental health in this population.

**Objectives:**

To determine the risk of depression and anxiety in adults with CP compared with an age-, sex-, and practice-matched reference group of adults without CP, using primary care data.

**Design, Setting, and Participants:**

Retrospective longitudinal cohort study set in UK primary care. Data were analyzed using Cox proportional hazards regression analyses adjusted for chronic conditions and visits to their physician. The study period ran from January 1987 to November 2015. Data of entry into the study ranged from January 1987 to September 2015. Data for 1705 adults 18 years or older with CP and 5115 matched adults without CP were extracted. Cerebral palsy was identified using diagnostic codes, and each person with CP was compared with 3 age-, sex-, and practice-matched controls.

**Exposures:**

Diagnosis of CP, with a second analysis accounting for comorbidity of intellectual disability (ID).

**Main Outcomes and Measures:**

Time to diagnosis for depression or anxiety following the date of entry into the study in adults with CP (with and without ID) compared with matched controls.

**Results:**

The mean (SD) age of the 1705 patients with CP and the 5115 adults without CP was 33.3 (15.5) years, and 798 participants (46.8%) were women. Individuals with CP had an increased adjusted hazard of depression (hazard ratio [HR], 1.28; 95% CI, 1.09-1.51) and anxiety (HR, 1.40; 95% CI, 1.21-1.63) compared with the matched reference group. When we accounted for ID comorbidity, there were 363 adults with CP who also had ID (mean [SD] age, 32.1 [13.2] years; 159 women [47.6%]) and 1342 adults with CP who did not have ID (mean [SD] age, 33.6 [16.1] years; 639 women [43.8%]). Only those individuals with CP and no comorbid ID had a higher risk of incident depression (HR, 1.44; 95% CI, 1.20-1.72) and anxiety (HR, 1.55; 95% CI, 1.28-1.87) than their matched controls.

**Conclusions and Relevance:**

Adults with CP have an increased risk of depression or anxiety. In particular, these results indicate that this association is driven largely by those individuals with CP with no co-occurring ID. Future work is needed in community-based samples to fully elucidate the causal mechanisms driving these associations.

## Key Points

**Questions:**

What is the risk of depression and anxiety in adults with cerebral palsy compared with age-, sex-, and practice-matched controls?

**Findings:**

In this cohort study of 1705 adults with cerebral palsy and a matched group of 5115 adults without cerebral palsy, adults with cerebral palsy had an increased risk of depression and anxiety compared with age-, sex-, and practice-matched controls. When intellectual disability comorbidity was accounted for, it was only those adults who had cerebral palsy with no intellectual disability who demonstrated an increased risk of depression and anxiety.

**Meaning:**

Adults with cerebral palsy have a higher risk of developing anxiety and depression than adults who do not have cerebral palsy.

## Teaser

This cohort study assesses depression and anxiety in adults with cerebral palsy compared with age-, sex-, and practice-matched adults with cerebral palsy.

## Introduction

*Cerebral palsy* (CP) is the umbrella term used for a heterogeneous group of etiologies that occur to a developing fetal or infant brain.^1^ People diagnosed as having CP have similar hallmark symptoms including issues with movement, coordination, posture, and balance.^1,2^ These motor disturbances can also be comorbid with other issues such as behavioral disturbance, cognitive difficulties, communication difficulties, sensory impairments, epilepsy, and intellectual disability (ID).^1^It is estimated that CP affects 2 to 3 in 1000 live births, and approximately 1 in 400 people in the United Kingdom live with CP.^3,4^

Because CP develops and is diagnosed in early childhood,^5^ it is often considered a pediatric condition. However, CP is a lifelong condition with the majority of children living into adulthood, depending on the severity of the condition and associated physical comorbidities.^6,7^ There is evidence that as individuals with CP transition into and throughout adulthood, there can be deterioration in physical functioning and a rise in secondary health conditions.^8,9^ The experience of aging with CP is therefore likely to be linked with different psychological, social, and medical issues than those experienced through having CP as a child. However, most evidence on mental health and CP is focused on children,^10^ which cannot be generalized to adult populations.

Depression and anxiety are 2 of the most common mental illnesses in the general population,^11^ and there is substantial evidence that living with a long-term condition or disability is associated with a 2- to 3-fold increase in the likelihood of being diagnosed as having depression^12,13^ or anxiety.^14^ However, there is relatively little research specifically examining mental health outcomes in adults with CP, to our knowledge.

Existing evidence indicates that 20% to 25% of adults with CP have clinically significant levels of depressive symptoms.^15,16^ Furthermore, a recent clinical study in 501 adults with CP from a US clinic found that 39% of patients met criteria for a diagnosis of anxiety disorder, and 31% met criteria for a diagnosis of major depression.^17^ A recent article that compared the prevalence of depression and anxiety in adults with CP to the general population found that adults with CP were more likely to experience depression, but not anxiety, than the general population.^18^ However, this work was cross sectional, and there is a need for longitudinal work to systematically investigate whether CP is associated with an increased risk of anxiety or depression in adulthood.

However, the association between depression, anxiety, and CP could be modified by comorbid ID. Approximately one-third of individuals with CP also experience comorbid ID^2^; however, ID has been associated with difficulties correctly identifying common mental illness owing to diagnostic overshadowing.^19^ Diagnostic overshadowing can happen in individuals with ID because distress (including anxiety and depression) can present as challenging behaviors instead of the symptoms we typically associate with depression and anxiety.^20,21^ In other words, the challenging behaviors overshadow the correct diagnosis of mental illness. This could mean that although we might expect the incidence of anxiety and depression to be higher in adults with CP, it is also important to account for the presence of ID.

The aim of this article was to determine the incidence of depression and anxiety in adults with CP compared with age-, sex-, and general practice–matched controls using primary care data from the United Kingdom. We also sought to determine whether the presence of comorbid ID impacted on the associations between CP and incident depression and anxiety.

## Methods

### Clinical Practice Research Datalink Database

Data for this study were taken from the Clinical Practice Research Datalink (CPRD) primary care database. This database reflects the collection of consultation data from consenting general practices throughout the United Kingdom, and it covers 6.9% of the UK population with active data available for 4.4 million people.^22^ Previous work has shown the population contained within the CPRD database are representative of the UK population.^22^ Collected data include clinical events, prescriptions, referrals, and hospital admissions. Formal data collection commenced in 1987, and data for this study were collected from January 1, 1987, to November 30, 2015. Data of entry into the study ranged from January 1987 to September 2015. Clinical Practice Research Datalink obtained ethical approval from a National Research Ethics Service Committee, which allows researchers to access anonymized data for observational studies on the approval of a protocol to an Independent Scientific Advisory Committee. Patient consent was waived.

### Participants

Any patient 18 years or older who had at least 1 record of CP during the study period, and during a period in which their data were considered research standard (ie, their data were determined by CPRD to be of sufficient quality for research assessment), was included as a case of CP. Clinical Practice Research Datalink checks data to ensure they are research standard by determining whether the patient-level data consist of valid registration dates and that the data provided by the practice have been continuous.^22^

Diagnoses were identified using Read codes, which are alphanumeric codes used in UK health care to reference a Read term that captures the reason for consultation (eg, the Read code F2B..00 refers to the Read term *cerebral palsy*) A diagnosis of CP was identified by 1 of 22 Read codes for CP that were created by the senior investigator (J.M.R.) and checked by other CP experts (eTable 1 in the Supplement). The index date, ie, the start of follow-up, was defined as the latest of^1^ the date that the patient registered with their general practitioner (GP),^2^ the date that their data became research standard,^3^ or the year in which they turned age 18 years. Initially, 14 788 patients with CP were identified. Following exclusions based on age (<18 years, n = 2510), a Read code for CP not occurring within the study period and/or a period when data were research standard (n = 10 038), and potentially inaccurate codes (n = 535), a sample of 1705 patients with CP were included within the main analysis.

Each patient with CP was matched to 3 patients without CP for age (within ±3 years), sex, and practice. Practice was used as an indicator of area-level socioeconomic status, as CPRD uses area-level deprivation as an indicator of socioeconomic position.^23^ In total, 5115 matched controls were identified, and these matched controls acted as the reference (comparison) group for our main analyses. The index date for each patient without CP was set as the same date as their respective matched CP case.

In additional analyses, we examined the association of ID comorbidity with the incidence of anxiety and depression in adults with CP. These cases were identified using a list of Read codes provided by the Cambridge primary care unit.^24^ For these additional analyses, we split the CP group into patients with CP with no ID comorbidity (CPnoID) and patients with CP and ID (CP+ID).

### Identification of Depression and Anxiety

Cases of depression and anxiety were identified using the Read codes developed by John et al^25^ and the Cambridge primary care unit.^24^ Where the codes could refer to a possible case of depression (eg, mood disorders, depression-related symptoms) or anxiety (eg, worrying), these were only considered a case of depression in which the patient was also given antidepressants and/or anxiolytic medication (eTable 1 in the Supplement includes a list of Read codes). We excluded those codes that referred to a history of depression or anxiety, depression, or anxiety remission, interim reviews, or medication reviews (as we wanted to ascertain the first event of depression and anxiety after the index date). The date of the first event of depression was identified following the index date. Where no event of depression occurred, participants were followed up until the earliest of the following and treated as censored: transfer out of CPRD, death, or end of follow-up period. This was repeated for anxiety.

### Identification of Intellectual Disability

Read codes developed by the Cambridge primary care unit^24^ were used to identify individuals with ID. These Read codes included specific conditions associated with ID such as fragile X syndrome and Down syndrome alongside other synonyms for “learning disability” and ”intellectual disability.”^24^

### Potential Confounders

As other chronic conditions and GP visits could be linked with an increased likelihood of detecting incident anxiety and depression, we adjusted our analyses for these confounders. We used the Read codes proposed by the Cambridge primary care unit^24^ to identify the following chronic conditions: heart disease (myocardial infarction, coronary heart disease, and/or arrhythmia), lung disease (chronic obstructive pulmonary disease, chronic bronchitis, and/or asthma), pain conditions (4 or more prescriptions of pain medication), epilepsy, and diabetes. We also identified Read codes for osteoarthritis based on *International Classification of Diseases, Ninth Revision*, and *International Statistical Classification of Diseases and Related Health Problems, Tenth Revision*, Read terms. Finally, we identified the mean number of GP visits per year, (0-2 visits per year, 2-11.9 visits per year, or ≥12 visits per year). We used 12 visits per year as an indicator for frequent GP consultations as has been done in previous work.^26^ For each potential confounder, we identified only those cases that occurred before the event date (ie, depression diagnosis or anxiety diagnosis) or the date of censoring.

### Statistical Analysis

To ascertain risk, we used stratified Cox proportional hazards regression for depression and anxiety as outcomes for the group with CP compared with the matched reference group. These were first run unadjusted and then adjusted for potential confounders.

In our stratified analysis, we then reran all analyses with the CP group stratified by the presence of comorbid ID. For these analyses, we compared CPnoID and CP+ID with their respective matched reference groups. Prior to running our analyses, we checked the assumption of proportional hazards by plotting scaled Schoenfeld residuals against time for all models and found this assumption was satisfied. All analyses were conducted using Stata, version 14.0 (StataCorp).

## Results

### Descriptive Data

We present descriptive data pertaining to the sociodemographic and health-related characteristics of the sample in Table 1. The mean (SD) age of the sample was 33.3 (15.5) years, and 798 individuals (46.8%) in both the group with CP and matched controls were women. The data also show that individuals with CP had a higher frequency of attending the physician, a higher frequency of epilepsy, and a higher frequency of pain conditions than the matched controls.

**Table 1.** Characteristics of Participants

Variable	No. (%)			
	No CP (n = 5115)	CP (n = 1705)	CPnoID (n = 1342)	CP+ID (n = 363)
Age, y				
<30	2631 (51.4)	877 (51.4)	691 (51.5)	186 (51.2)
30-39	1008 (19.7)	335 (19.7)	252 (18.8)	84 (23.1)
40-49	669 (13.1)	223 (13.1)	172 (12.8)	51 (14.1)
50-59	405 (7.9)	135 (7.9)	108 (8.1)	27 (7.4)
≥60	402 (7.9)	134 (7.9)	119 (8.9)	15 (4.1)
Sex				
Male	2721 (53.2)	907 (53.2)	703 (52.8)	204 (56.2)
Female	2394 (46.8)	798 (46.8)	639 (47.6)	159 (43.8)
Region				
North	1419 (27.7)	473 (27.7)	349 (26.0)	124 (34.2)
Midlands	1809 (35.4)	603 (35.4)	498 (37.1)	105 (28.9)
South	1761 (34.4)	587 (34.4)	459 (34.0)	128 (35.3)
Northern Ireland	126 (2.5)	42 (2.5)	36 (2.7)	6 (1.7)
GP visits per year, mean (SD)				
0-1.9	715 (14.0)	133 (7.8)	123 (9.2)	10 (2.8)
2-11.9	4035 (79.0)	1178 (69.3)	956 (71.5)	222 (61.3)
≥12	358 (7.0)	388 (22.8)	258 (19.3)	130 (35.9)
Presence of				
Depression	867 (17.0)	312 (18.3)	263 (19.6)	49 (13.5)
Anxiety	697 (13.6)	261 (15.3)	216 (16.1)	45 (12.4)
Diabetes	218 (4.3)	55 (3.2)	47 (3.5)	14 (3.9)
Heart disease	584 (11.4)	160 (9.4)	165 (12.3)	20 (5.5)
Osteoarthritis	320 (6.3)	87 (5.1)	90 (6.7)	15 (4.1)
Epilepsy	49 (1.0)	354 (20.8)	216 (16.1)	155 (42.7)
Lung disease	379 (7.4)	147 (8.6)	143 (10.7)	26 (7.2)
Pain conditions	238 (4.7)	166 (9.7)	179 (13.3)	23 (6.3)

Abbreviations: CP, cerebral palsy; CPnoID, cerebral palsy with no comorbid ID; CP+ID: cerebral palsy with comorbid ID; GP, general practitioner; ID, intellectual disability.

### Risk of Depression

During the follow-up period, there were 1179 new events of depression following the index date. In total, 867 people (17.0%) from the reference group had a new event of depression during a median of 9.1 (range, 0.01-28.01) years of follow-up. In total, 312 patients (18.3%) with CP had a new event of depression during a median of 5.7 (range, 0.001-27.9) years of follow-up (Table 2). The unadjusted Cox model indicated that patients with CP had an increased hazard of depression compared with matched patients without CP (hazard ratio [HR], 1.43; 95% CI, 1.24-1.64). This association remained statistically significant (HR, 1.28; 95% CI, 1.09-1.51) after controlling for other chronic conditions and the mean number of GP visits (Table 2).

**Table 2.** Incidence of Depression and Anxiety in 1705 Individuals With CP Compared With 5115 Age-, Sex-, and Practice-Matched Controls

Variable	Events No. (%)	Person-Years in 10 000s	Incidence Per 10 000 Person-Years (95% CI)	Unadjusted		Adjusted	
				Hazards Ratio (95% CI)	*P* Value	HR (95% CI)^a^	*P* Value
Depression							
No CP	867 (17.0)	49.93	0.017 (0.016-0.019)	1 [Reference]	NA	1 [Reference]	NA
CP	312 (18.3)	12.64	0.025 (0.022-0.028)	1.43 (1.24-1.64)	<.001	1.28 (1.09-1.51)	.003
Anxiety							
No CP	697 (13.6)	51.67	0.013 (0.013-0.015)	1 [Reference]	NA	1 [Reference]	NA
CP	261 (15.3)	12.93	0.020 (0.018-0.023)	1.40 (1.21-1.63)	<.001	1.38 (1.15-1.64)	<.001

Abbreviations: CP, cerebral palsy; NA, not applicable.

^a^ Adjusted for baseline (ie, predepression or preanxiety diagnosis) diagnosis of diabetes, heart disease, lung disease, osteoarthritis, epilepsy, pain conditions, and general practitioner visits per year.

After stratifying for the presence of comorbid ID in individuals with CP, we found that there were 264 new cases (19.6%) of depression in the CPnoID group during a median of 5.2 (range, 0.05-27.9) years of follow-up. However, there were 48 incident cases (13.3%) of depression in the CP+ID group during a median of 7.4 (range, 0.01-26.1) years of follow-up. When we conducted Cox regression analysis, stratified according to presence of ID, we found that the CPnoID group had an increased adjusted hazards of incident depression compared with their matched reference group (adjusted HR, 1.44; 95% CI, 1.20-1.72) (Table 3). For both unadjusted and adjusted analyses, the CP+ID group had no difference in their hazard of incident depression compared with their matched reference group (Table 3). For Kaplan-Meier plots, see the eFigure in the Supplement.

### Risk of Anxiety

During the follow-up period, there were 958 new events of anxiety after the index date. In total, 697 people (13.6%) from the reference group had a new event of anxiety during a median of 9.5 (range, 0.01-28.0) years of follow-up and 261 patients (15.3%) with CP had a new event of anxiety during a median of 6.0 (range, 0.003-27.9) years of follow-up (Table 3). The unadjusted HR indicated an increased risk of anxiety for individuals with CP compared with the matched reference group (HR, 1.40; 95% CI, 1.21-1.63). This increased risk persisted after controlling for other chronic conditions and the mean number of GP visits (HR, 1.38; 95% CI, 1.15-1.64) (Table 3).

**Table 3.** Incidence of Depression and Anxiety in Individuals With CP With and Without Comorbid ID^a^

Variable^b^	Events, No. (%)	Person-Years in 10 000s	Incidence Per 10 000 Person-Years (95% CI)	Unadjusted		Adjusted	
				Hazard Ratio (95% CI)	*P* Value	Hazard Ratio (95% CI)^c^	*P* Value
Depression							
Matched reference group	687 (17.0)	39.32	0.017 (0.016-0.019)	1 [Reference]	NA	1 [Reference]	NA
CPnoID	264 (19.6)	9.55	0.028 (0.025-0.031)	1.59 (1.36-1.85)	<.001	1.44 (1.20-1.72)	<.001
Depression							
Matched reference group	180 (16.6)	10.61	0.017 (0.015-0.020)	1 [Reference]	NA	1 [Reference]	NA
CP+ID	48 (13.3)	3.10	0.015 (0.012-0.021)	0.92 (0.66-1.29)	.66	0.68 (0.43-1.07)	.09
Anxiety							
Matched reference group	542 (13.4)	40.72	0.013 (0.012-0.014)	1 [Reference]	NA	1 [Reference]	NA
CPnoID	217 (16.2)	9.79	0.022 (0.019-0.025)	1.57 (1.32-1.85)	<.001	1.55 (1.28-1.87)	<.001
Anxiety							
Matched reference group	155 (14.3)	10.94	0.014 (0.012-0.017)	1 [Reference]	NA	1 [Reference]	NA
CP+ID	44 (12.2)	3.15	0.014 (0.010-0.019)	0.92 (0.65-1.30)	.65	0.77 (0.48-1.25)	.29

Abbreviations: CP, cerebral palsy; CPnoID, cerebral palsy with no comorbid ID; CP+ID, cerebral palsy with comorbid ID; ID, intellectual disability; NA, not applicable.

^a^ Each CP group was compared with their respective age-, sex-, and practice-matched reference group. There were 1342 individuals in the CPnoID group and 4026 in their respective matched group. There were 363 individuals in the CP+ID group and 1089 in their respective matched group.

^b^ CPnoID compared with respective matched controls and CP+ID compared with respective matched controls.

^c^ Adjusted for baseline (ie, predepression or preanxiety diagnosis) diagnosis of diabetes, heart disease, lung disease, osteoarthritis, epilepsy, pain conditions, and general practitioner visits per year.

After stratifying for comorbid ID, we found that in the CPnoID group, there were 217 new cases (16.2%) of incident anxiety during a median of 5.6 (range, 0.003-27.9) years of follow-up (Table 3). In the CP+ID group, there were 44 cases (12.2%) of incident anxiety during a median of 7.7 (range, 0.06-26.1) years of follow-up (Table 3). The Cox regression analysis indicated that the CPnoID group had an increased adjusted hazards of incident anxiety compared with their matched reference group (HR, 1.55; 95% CI, 1.28-1.87) (Table 3). However, the CPnoID group had no difference in their unadjusted or adjusted hazards of experiencing incident anxiety compared with their matched reference group (Table 3). For Kaplan-Meier plots, see the eFigure in the Supplement.

### Sensitivity Analysis

As a total of 24 participants in the no-CP group also had ID, which could affect estimates, we reran all analyses excluding these participants from our ID-stratified analyses (eTable 2 in the Supplement). Removal of individuals with ID from the control group did not have a substantial effect on the results (eTable 2 in the Supplement).

## Discussion

Results from this study indicated that individuals with CP had an increased risk of being diagnosed as having depression or anxiety, compared with a matched control group of adults without CP. These results could have been observed because adults with CP present with many physiological, psychological, social, and health-related risk factors that have been shown to be associated with depression and anxiety in the general population such as multimorbidity,^27,28,29^ increased pain,^30,31,32^ functional limitations,^2,33^ noncommunicable diseases,^12,14,34,35^ difficulties with social relationships^36,37^ and poorer sleep.^38,39^ Furthermore, when we examine work that has been conducted in adults with CP, depressive symptoms are associated with fatigue^15^ and pain.^15,40^ However, we need more research to determine why individuals with CP may have a higher risk of depression and anxiety so that we may develop the evidence base for mental health interventions in this population. Our results also indicate that ID comorbidity should be considered when assessing the mental health of adults with CP. We found the risk of depression and anxiety was higher in the adults with CP who did not have ID, compared with the matched reference group. Furthermore, adults with CP and ID had similar hazards of developing depression and anxiety to the matched reference group. We could have observed these results as previous work suggests that diagnostic overshadowing may lead to an underdiagnosis of mental illness among people who have ID^19^ because distress can present as challenging behaviors.^20,21^ Thus, it is possible that GPs may not be as well trained in diagnosing depression and anxiety in these individuals. However, there is also work that has been conducted in populations with ID, indicating that the prevalence of anxiety and depression in individuals with ID is no different from the general population.^21^

While our evidence suggests an increased risk of developing anxiety and depression in adults with CP compared with adults who do not have CP, it should be noted that there was little difference in absolute risk for developing anxiety or depression over the total follow-up period.

This is the first study, to our knowledge, to compare the risk of depression and anxiety in adults with CP to adults who do not have CP using a population-based cohort. The results support previous work that shows a relatively high frequency of depression and anxiety in individuals with CP.^15,17^

### Limitations

Owing to the nature of the population examined and the reliance on the presence of diagnostic codes to define outcomes, there is a possibility that any observed associations are underestimated as previous work suggests depression and anxiety diagnoses in primary care may be underestimated.^41,42^ This could also explain why the rates of depression and anxiety we observed in individuals with CP for this study were lower than other studies have previously reported.^17,18^ Furthermore, *CP* is an umbrella term used to describe heterogeneous etiologies; however, we could not account for the severity of issues associated with CP. These include gross motor function, communication issues, subtypes of motor abnormality, and fatigue. Future work should provide more insight into how CP-specific issues might be associated with mental health. In addition, the measure of pain conditions that we had within this study relied only on medications.

There are also additional caveats that should be borne in mind when interpreting these data. Depression and anxiety are considered chronic conditions that typically have their first onset in adolescence or early adulthood^43^; therefore, it is plausible to assume that some people may have had diagnoses of depression and anxiety prior to the index date for this study. In future work, it could be interesting to look at the life course of mental illness in individuals with CP, looking at people from adolescence through to older age.

## Conclusions

This work provides evidence that adults with CP have an increased risk of developing depression and anxiety. Furthermore, comorbidity of ID is an important effect modifier that should be considered when examining the mental health of adults with CP. There is a need for more work to elucidate the causal mechanisms of poor mental health in adults with CP so that we may develop targeted interventions.

## Notes

Supplement. eTable 1. Read codes and associated Read terms used to define Cerebral Palsy eTable 2. Risk of depression and anxiety in individuals with CP with and without co-morbid ID (excluding all individuals without CP who had a diagnosis of ID, n=24) eFigure. Kaplan Meier survival plots
