# Supplementary material for: Risk of Depression and Anxiety in Adults With Cerebral Palsy
Source: JAMA Neurol. 2018 Dec 28;76(3):294–300. doi: 10.1001/jamaneurol.2018.4147 (PMC6439715; doi:10.1001/jamaneurol.2018.4147)
Supplement: Supplement. — eTable 1. Read codes and associated Read terms used to define Cerebral Palsy eTable 2. Risk of depression and anxiety in individuals with CP with and without co-morbid ID (excluding all individuals without CP who had a diagnosis of ID, n=24) eFigure. Kaplan Meier survival plots [file jamaneurol-76-294-s001.pdf]

Supplementary Online Content

Smith KJ, Peterson MD, O'Connell NE, et al. Risk of depression and anxiety in adults with cerebral palsy. *JAMA Neurol*. Published online December 28, 2018. doi:10.1001/jamaneurol.2018.4147

eTable 1. Read codes and associated Read terms used to define Cerebral Palsy

eTable 2. Risk of depression and anxiety in people with CP with and without co-morbid ID (excluding all people without CP who had a diagnosis of ID, n=24)

eFigure. Kaplan Meier survival plots

This supplementary material has been provided by the authors to give readers additional information about their work.

© 2018 Smith KJ et al. *JAMA Neurology.*

eTable 1. Read codes and associated Read terms used to define Cerebral Palsy

**Read code**	**Read term**
F23y400	Ataxic diplegic cerebral palsy
F23y000	Ataxic diplegic cerebral palsy
F137.11	Athetoid cerebral palsy
F137000	Athetoid cerebral palsy
F2B..00	Cerebral palsy
F2Bz.00	Cerebral palsy NOS
F230100	Cerebral palsy with spastic diplegia
F23..00	Congenital cerebral palsy
F23y300	Dyskinetic cerebral palsy
F23..12	Infantile cerebral palsy
F23y200	Spastic cerebral palsy
F230111	Spastic diplegic cerebral palsy
F2B1.00	Spastic hemiplegic cerebral palsy
F2B0.00	Spastic quadriplegic cerebral palsy
F23yz00	Other infantile cerebral palsy NOS
Fyu9000	[X]Other infantile cerebral palsy
F23y.00	Other congenital cerebral palsy
F23y100	Flaccid infantile cerebral palsy
F2By.00	Other cerebral palsy
Fyu9.00	[X]Cerebral palsy and other paralytic syndromes
F23z.00	Congenital cerebral palsy NOS
F23..11	Congenital spastic cerebral palsy
F23y600	Choreoathetoid cerebral palsy

© 2018 Smith KJ et al. *JAMA Neurology.*

Read codes and associated Read terms used to define Depression

Read Code	Read term
**Depression**	
Eu32700	[X] Major depression, severe without psychotic symptoms
Eu32200	[X] Severe depressive episode without psychotic symptoms
Eu32213	[X] Single episode vital depression without psychotic symptoms
Eu33214	[X] Vital depression, recurrent without psychotic symptoms
Eu3y111	[X] Recurrent brief depressive episodes
E113700	Recurrent depression
E2B1.00	Chronic depression
E113200	Recurrent major depressive episodes, moderate
E113.00	Recurrent major depressive episode
Eu33100	[X] Recurrent depressive disorder, current episode moderate
E112.11	Agitated depression
E135.00	Agitated depression
Eu32.11	[X] Single episode of depressive reaction
E112200	Single major depressive episode, moderate
Eu33211	[X] Endogenous depression without psychotic symptoms
Eu32.13	[X] Single episode of reactive depression
E112300	Single major depressive episode, severe, without psychosis
E112000	Single major depressive episode, unspecified
Eu33200	[X] Recurrent depressive disorder current episode severe without psychotic symptoms
E112.13	Endogenous depression first episode
E112.14	Endogenous depression
Eu33212	[X] Major depression, recurrent without psychotic symptoms
Eu32212	[X] Single episode major depression without psychotic symptoms
Eu32211	[X] Single episode agitated depression without psychotic symptoms
Eu33000	[X] Recurrent depressive disorder, current episode mild
E113700	Recurrent depression
Eu33.11	[X] Recurrent episodes of depressive reaction
E11y200	Atypical depression disorder
E113100	Recurrent major depression episodes, mild
Eu32400	[X] Mild depression
Eu32z13	[X] Prolonged single episode of reactive depression
E113.11	Endogenous depression - recurrent
E112.12	Endogenous depression first episode
E112.00	Single major depression episode
E290.00	Brief depressive reaction
Eu32y11	[X] Atypical depression
Eu33.13	[X] Recurrent episodes of reactive depression
E113300	Recurrent major depressive episodes, severe, no psychosis

© 2018 Smith KJ et al. *JAMA Neurology.*

Eu33.12	[X] Recurrent episodes of psychogenic depression
Eu32.12	[X] Single episode of psychogenic depression
Eu32600	[X] Major depression, moderately severe
Eu33.00	[X] Recurrent depressive disorder
Eu33z00	[X] Recurrent depressive disorder, unspecified
E291.00	Prolonged depressive reaction
Eu32500	[X] Major depression, mild
E112100	Single major depressive episode, mild
Eu32000	[X] Mild depressive episode
Eu32.00	[X] Depressive episode
Eu32z00	[X] Depressive episode, unspecified
E2B..00	Depressive disorder NEC
1B17.00	Depressed
2257	o/e - depressed
1B1U.11	Depressive symptoms
1B1U.00	Symptoms of depression
1B17.11	c/o - feeling depressed
1BT..00	Depressed mood
1JJ..00	Suspected depression
**Depression other or NOS**	
E113z00	Recurrent major depressive episode nos
Eu33y00	[X] Other recurrent depressive disorders
Eu32y00	[X] Other depressive episodes
E113000	Recurrent major depressive episodes, unspecified
E290z00	Brief depressive episode nos
Eu32y12	[X] Single episode of masked depression NOS
E11z200	Masked depression
Eu33z11	[X] Monopolar depression NOS
Eu32z14	[X] Reactive depression NOS
E112z00	Single major depression episode NOS
Eu32100	[X] Moderate depressive episode
Eu32z11	[X] Depression NOS
Eu32z12	[X] Depressive disorder NOS
E112.11	agitated depression
Eu32211	[x]single episode agitated depression w'out psychotic symptoms
**Dysthymia**	
Eu34100	Dysthymia
Eu34112	[X] Depressive personality disorder
E211200	Depressive personality disorder
Eu34111	[X] Depressive neurosis
Eu34113	[X] Neurotic depression
E204.00	Neurotic depression reactive type
**Mixed depression anxiety**	
Eu41200	[X]Mixed anxiety and depressive disorder

© 2018 Smith KJ et al. *JAMA Neurology.*

Eu41211	[X]Mild anxiety depression
E200300	Anxiety with depression
Eu34114	[X] Persistant anxiety depression
**Secondary depression**	
E204.11	Postnatal depression
Eu53011	[X] Postnatal depression NOS
Eu32B00	[X] Antenatal depression
E001300	Presenile depression with dementia
E002100	Senile dementia with depression
Eu53012	[X] Postpartum depression nos
Eu02z16	[X] Senile dementia, depressed or paranoid type
E002.00	Senile dementia with depressive or paranoid features
R007z13	[d] postoperative depression
E002z00	Senile dementia with depressive or paranoid features NOS
62T1.00	Puerperal depression
E004300	Arteriosclerotic dementia with depression
Eu20400	[x] Post-schizophrenic depression
E02y300	Drug-induced depressive state
Eu92000	[X] Depressive conduct disorder
Eu33.15	[X] SAD- seasonal affective disorder
E118.00	Seasonal affective disorder
Eu33.14	[X] Seasonal depressive disorder
**Therapy**	
8CAa.00	Patient given advice about management of depression
8HHq.00	Referral for guided self-help for depression

© 2018 Smith KJ et al. *JAMA Neurology.*

Drugs used to identify possible depression (to be considered a case of depression patients were required to also have one of the read codes indicative of possible depression)

**Antidepressants**		
		Dosulepin
		Imipramine
		Lofepramine
		Nortriptyline
		Trimipramine
		Amoxapine
		Dothiepin
		Maprotiline
		Mianserin
		Trazadone
**Antidepressants (MAOIs)**		
		Phenelzine
		Isocarboxazid
		Tranlcypromine
		Moclobemide
**Antidepressant (SSRI)**		
		Citralopram
		Fluoxetine
		Fluvoxamine
		Paroxetine
		Sertraline
		Escitalopram
**Antidepressant (other)**		
		Flupentixol
		Mirtazapine
		Reboxetine
		Venlafaxine
		Tryptophan
		Agomelatine
		Duloxetine

© 2018 Smith KJ et al. *JAMA Neurology.*

Read codes and associated Read terms used to define possible depression.

Read code	Read term
1BT..11	Low mood
1B1J.11	Emotional upset
16ZB100	feeling low or worried
7899	o/e - distressed
E205.11	nervous exhaustion
E20z.11	nervous breakdown
E290011	bereavement reaction
E290000	Grief reaction
8HHK.00	referral to bereavement counsellor
IBT..12	sad mood
1BT..11	Low mood
Eu3z.00	[X] Unspecified mood affective disorder
Eu05300	[X] Organic mood [affective] disorders
Eu34z00	[X] Persistant mood affective disorder, unspecified
Eu34.00	[X] Persistant mood affective disorders
Eu34y00	[X] Other persistant mood affective disorders
Eu3..00	[X] Mood - affective disorders
Eu3y.00	[X] Other mood affective disorders
Eu3y100	[X] Other recurrent mood affective disorders
Eu3y000	[X] Other single mood affective disorders
Eu3yy00	[X] Other specified mood affective disorders
1BO..00	Mood swings

© 2018 Smith KJ et al. *JAMA Neurology.*

Read codes and associated Read terms used to define Anxiety Disorders

Anxiety disorders were defined as generalised anxiety disorder, mixed depression and anxiety disorder, panic disorder or phobias. As obsessive-compulsive disorders and post-traumatic and acute stress disorders are no longer considered anxiety disorders within the DSM-V we opted to exclude these events as cases of anxiety.

**Read code**	**Read term**
**Mixed depression anxiety**	
Eu41200	[X]Mixed anxiety and depressive disorder
Eu41211	[X]Mild anxiety depression
E200300	Anxiety with depression
Eu34114	[X] Persistant anxiety depression
**Anxiety disorder diagnoses**	
Eu41.00	[x] other anxiety disorders
Eu40y00	[x]other phobic anxiety disorders
Eu41000	[x]panic disorder [episodic paroxysmal anxiety]
Eu40012	[x] panic disorder with agoraphobia
1B1V.00	c/o - panic attack
Eu41011	[x] panic attack
225J.00	o/e - panic attack
E200111	panic attack
Eu41100	[x]generalized anxiety disorder
Eu41300	[X]Other mixed anxiety disorders
Eu41y00	[X]Other specified anxiety disorders
Eu41z00	[x]anxiety disorder, unspecified
Eu05400	[x]organic anxiety disorder
E2000000	Anxiety state unspecified
Eu41113	[x] anxiety state
E200100	panic disorder
Eu41100	[X] Generalized anxiety disorder
E200200	Generalised anxiety disorder
Eu41111	[x] anxiety neurosis
E200400	Chronic anxiety
E200500	Recurrent anxiety
E200.00	anxiety states
E200z00	anxiety state nos
Eu41z11	[x] anxiety nos
E292000	Separation anxiety disorder
Eu93000	[x] separation anxiety disorder to childhood
E2D0000	childhood and adolescent overanxiousness disturbance
E2D0.00	disturbance of anxiety and fearfulness childhood/ adolescent
E2D0z00	disturbance anxiety and fearfulness childhood/adolescent nos
Eu93y12	[x] childhood overanxious disorder

© 2018 Smith KJ et al. *JAMA Neurology.*

Eu41112	[x] anxiety reaction
1B13.00	anxiousness
2258	O/E - anxious
1B13.12	anxious
8G94.00	anxiety management training
8HHp.00	referral for guided self-help for anxiety
Z481.00	phobia counselling
8CAZ000	patient given advice about management of anxiety
8G52.00	antiphobic therapy
1B13.11	anxiousness - symptom
Z4I7.00	acknowledging anxiety
Z4I7100	recognising anxiety
Eu41012	[x]panic state
Eu41112	[x] anxiety reaction
**Phobias**	
Eu40000	[x]agoraphobia
Eu40011	[x]agoraphobia without history of panic disorder
E202100	agoraphobia with panic attacks
E202200	agoraphobia without mention of panic attacks
Eu40200	[x] specific (isolated) phobias
Eu40214	[x] simple phobia
E202700	animal phobia
E202C00	dental phobia
Eu40212	[x]animal phobias
E28z.12	flying phobia
E202A00	Fear of flying
E202E00	Fear of pregnancy
Eu40213	[x] claustrophobia
E202800	claustrophobia
Eu40300	[x]needle phobia
E202600	acrophobia
E202D00	Fear of death
Eu40100	[x]social phobias
E202400	social phobia, fear of public speaking
E202B00	cancer phobia
E202300	social phobia, fear of eating in public
E202500	social phobia, fear of public washing
E202.11	social phobic disorders
Eu93200	[x] social anxiety disorder of childhood
Eu93100	[x] phobic anxiety disorder of childhood
Eu45215	[x]nosophobia
Eu40211	[x] acrophobia
E202600	acrophobia
E202000	Phobia

© 2018 Smith KJ et al. *JAMA Neurology.*

Eu40z11	[x]phobia nos
E202.00	phobic disorders
E202z00	phobic disorder nos
E202.12	phobic anxiety
Eu40z00	[x] phobic anxiety disorder, unspecified
E202000	Phobia unspecified
Eu40y00	[x] other phobic anxiety disorders
Eu40.00	[x] phobic anxiety disorders
E292000	separation anxiety disorder
Eu40z12	[x] phobic state nos

© 2018 Smith KJ et al. *JAMA Neurology.*

Drugs used to identify possible anxiety (to be considered a case of anxiety patients were required to also have one of the read codes indicative of possible anxiety)

**Anxiolytics**		
		Diazepam
		Alprazolam
		Bromazepam
		Chlordiazepoxide
		Chlorezanone
		Clobazem
		Clorazepate dipotassium
		Hydroxyzine hcl (anxiolytic)
		Ketazolam - discontinued
		Lorazepam (anxiolytic)
		Medazepam - discontinued
		Meprobamate
		Oxazepam
SSRIs		Citralopram
		Fluoxetine
		Fluvoxamine
		Paroxetine
		Sertraline
		Escitalopram

Read codes and associated Read terms used to define possible anxiety.

**Read code**	**Read term**
1B12.00	Nerves' - nervousness
R2y2.00	(d) nervousness
R2y2.	O/E nervous
1B12.12	tension - nervous
R2y2.12	[d] nervous tension
E205.11	nervous exhaustion
E20z.11	nervous breakdown
1B1..00	general nervous symptoms
1B1Z.00	general nervous symptoms nos
2253	o/e - distressed
16ZB100	feeling low or worried
1BK..00	worried

© 2018 Smith KJ et al. *JAMA Neurology.*

eTable 2. Risk of depression and anxiety in people with CP with and without co-morbid ID (excluding all people without CP who had a diagnosis of ID, n=24).

		Events n (%)	*Person years in 10,000s*	*Incidence per 10,000 person years*	*Unadjusted hazards ratio (95% CI) and p-value*	*Adjusted hazards ratio (95% CI) †* and p-value
**Depression CPnoID**	***Matched reference group***	684 (17.02%)	39.22	0.017 (0.016-0.019)	1	1
	CPnoID	264 (19.64%)	9.55	0.028 (0.025-0.031)	1.59 (1.36-1.85), p<.001	1.43 (1.20-1.71), p<.001
**Depression CP+ID**	***Matched reference group***	177 (16.50%)	10.54	0.017 (0.014-0.019)	1	1
	CP+ID	48 (13.30%)	3.10	0.015 (0.012-0.021)	0.92 (0.66-1.28), p=.63	0.69 (0.43-1.09), p=.11
**Anxiety CPnoID**	***Matched reference group***	540 (13.44%)	40.61	0.013 (0.012-0.014)	1	1
	CPnoID	217 (16.15%)	9.79	0.022 (0.019-0.025)	1.57 (1.33-1.86), p<.001	1.56 (1.28-1.88), p<.001
**Anxiety CP+ID**	***Matched reference group***	152 (14.17%)	10.87	0.014 (0.012-0.016)	1	1
	CP+ID	44 (12.19%)	3.15	0.014 (0.010-0.019)	0.93 (0.65-1.31), p=.66	0.79 (0.48-1.27), p=.33

† Adjusted for baseline (i.e., pre-depression or pre-anxiety diagnosis) diagnosis of diabetes, heart disease, lung disease, osteoarthritis, epilepsy, pain conditions and GP visits per year.

CPnoID: Cerebral Palsy with no co-morbid ID

CP+ID: Cerebral Palsy with co-morbid ID.

Each CP group was compared to their respective age, sex and practice matched reference group. CPnoID N=1342 and their respective matched group N=4026. CP+ID N=363 and their respective matched group N=1089

© 2018 Smith KJ et al. *JAMA Neurology.*

eFigure. Kaplan Meier survival plots

Comparison of all people with CP (n=1,705) and all matched controls (n=5,115)

Graph i: Time to depression for people with CP and matched-controls

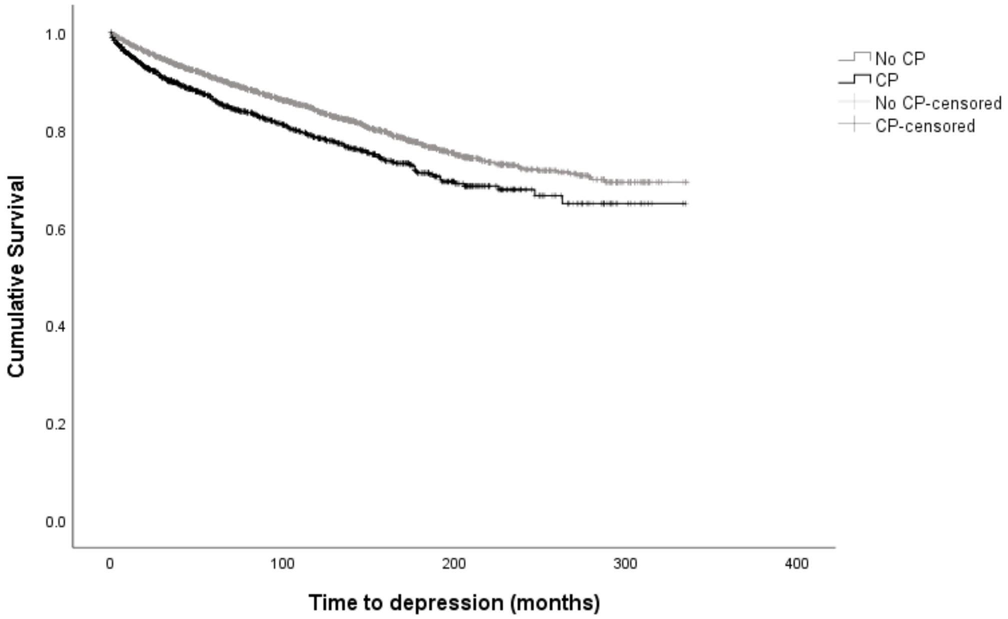

The number of people at risk at each time point were: 0 (n=6820), 100 (n=3998), 200 (n=932) and 300 (n=94).

Graph ii: Time to anxiety for people with CP and matched-controls

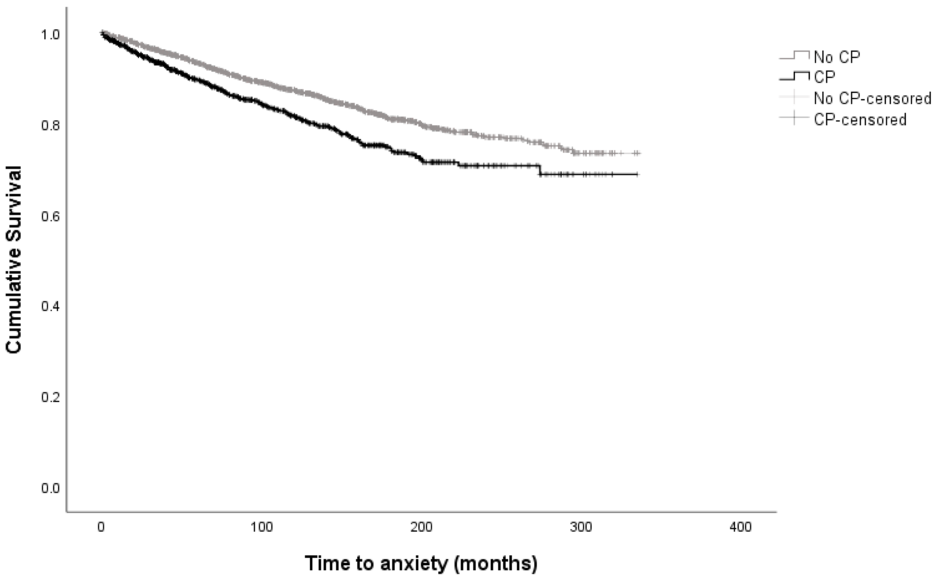

The number of people at risk at each time point were: 0 (n=6820), 100 (n=3507), 200 (n=993) and 300 (n=106).

© 2018 Smith KJ et al. *JAMA Neurology.*

Comparison of CPnoID (n=1,342) with respective matched controls (n=4,026)

Graph iii: Time to depression for people with CPnoID and respective matched-controls

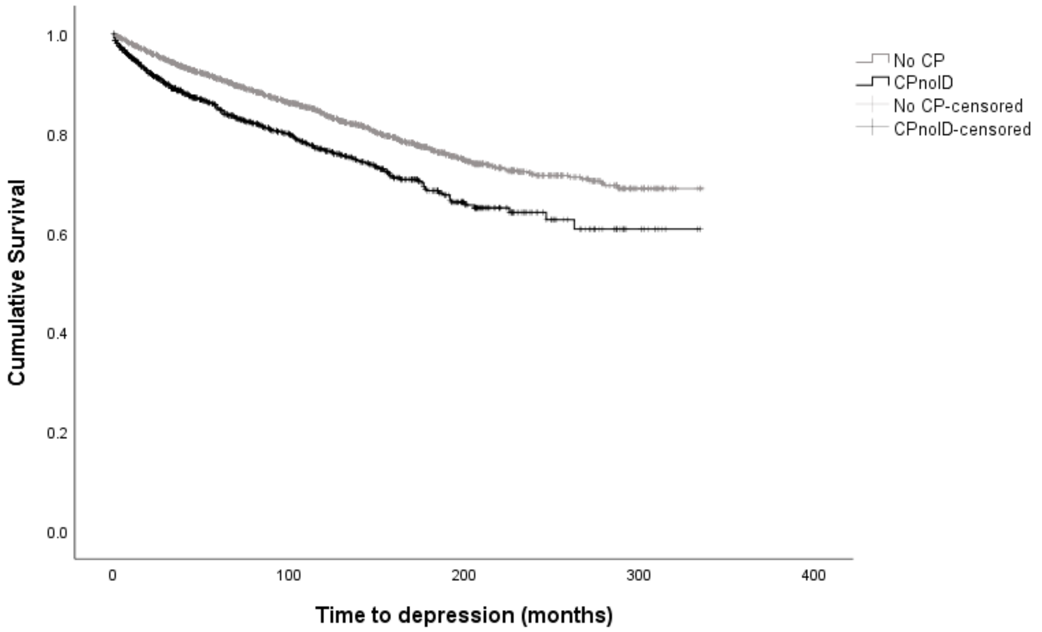

The number of people at risk at each time point were: 0 (n=5376), 100 (n=2632), 200 (n=737) and 300 (n=83).

Graph iv: Time to anxiety for people with CPnoID and respective matched-controls

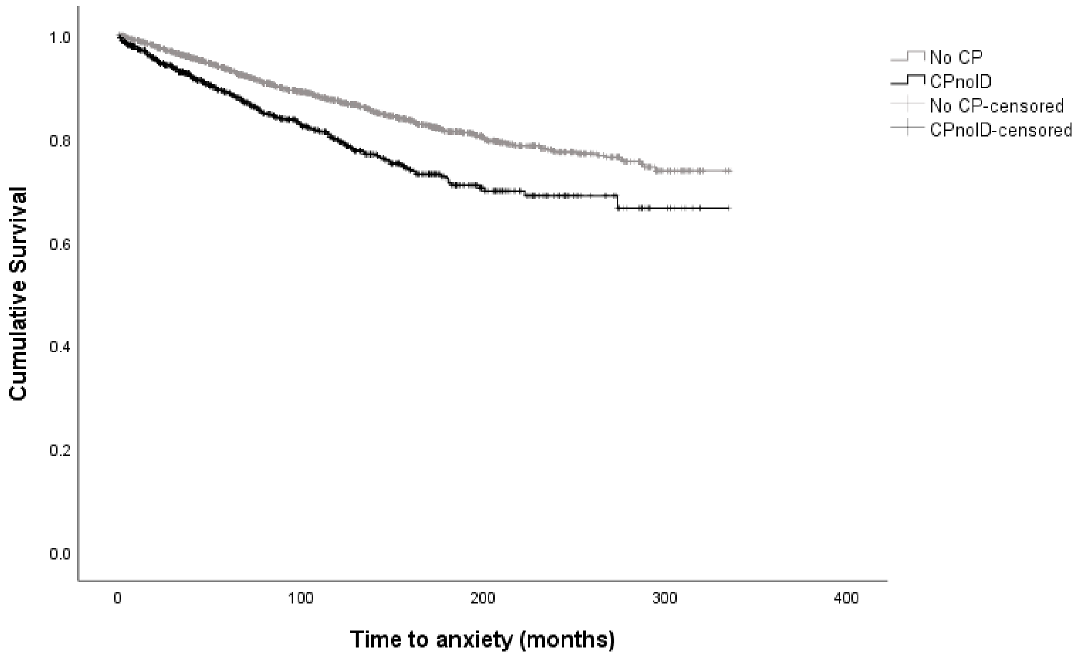

The number of people at risk at each time point were: 0 (n=5376), 100 (n=2714), 200 (n=798) and 300 (n=92).

© 2018 Smith KJ et al. *JAMA Neurology.*

Comparison of CP+ID (n=363) with respective matched controls (n=1,089)

Graph v: Time to depression for people with CP+ID and respective matched-controls

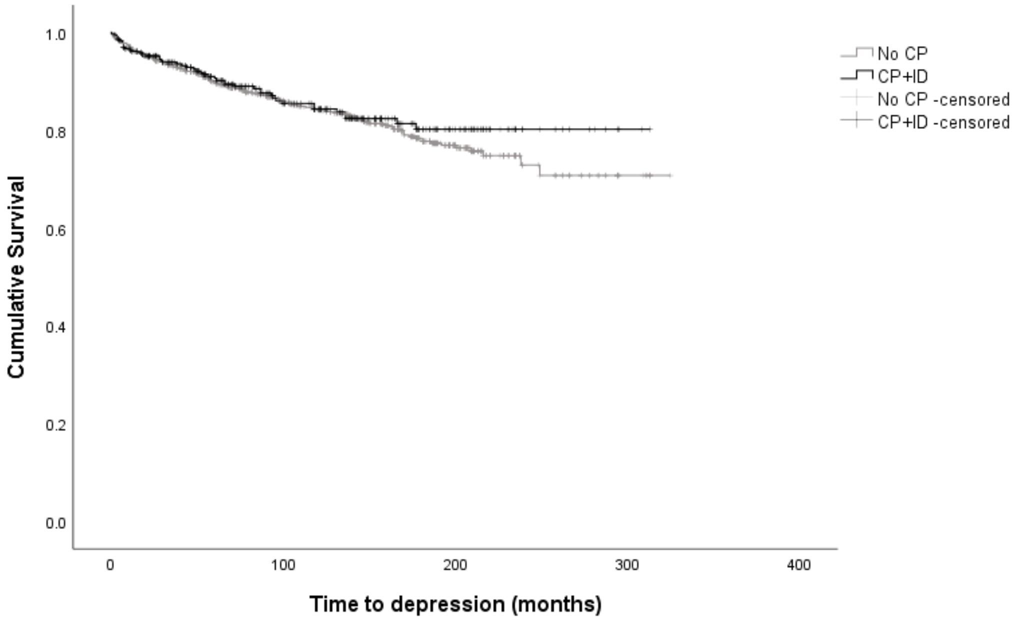

The number of people at risk at each time point were: 0 (n=1083), 100 (n=605), 200 (n=150) and 300 (n=9).

Graph vi: Time to anxiety for people with CP+ID and respective matched-controls

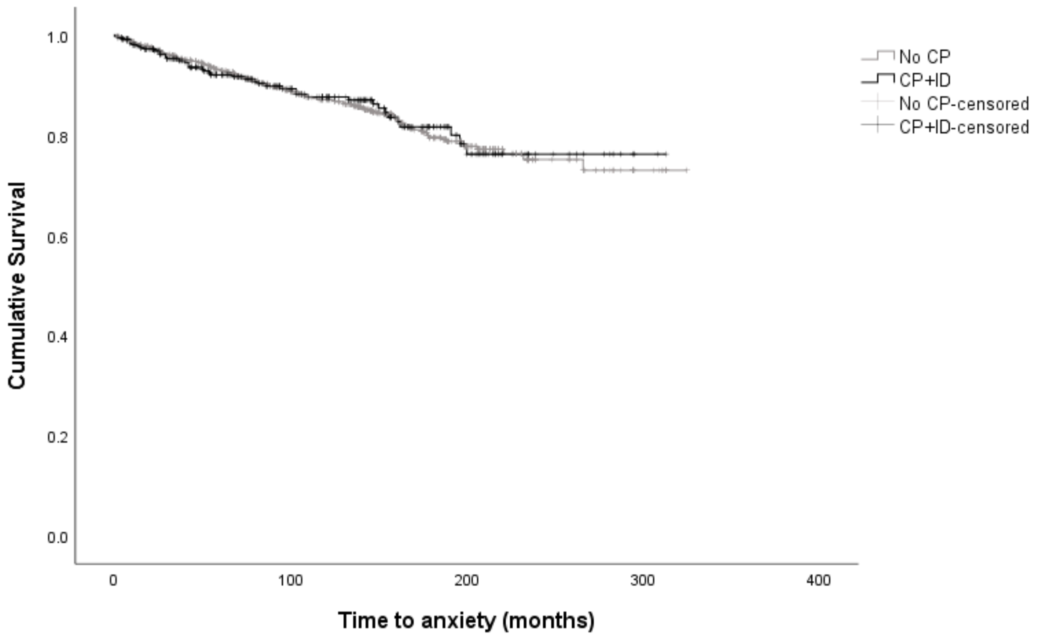

The number of people at risk at each time point were: 0 (n=1083), 100 (n=625), 200 (n=156) and 300 (n=12).

© 2018 Smith KJ et al. *JAMA Neurology.*
